# Is the Frequency Content of the Calls in North American Treefrogs Limited by Their Larynges?

**DOI:** 10.1155/2014/198069

**Published:** 2014-09-23

**Authors:** Marcos Gridi-Papp

**Affiliations:** Department of Biological Sciences, University of the Pacific, 3601 Pacific Avenue, Stockton, CA 95211, USA

## Abstract

A high diversity of mating calls is found among frogs. The calls of most species, however, are simple, in comparison to those of mammals and birds. In order to determine if the mechanics of the larynx could explain the simplicity of treefrog calls, the larynges of euthanized males were activated with airflow. Laryngeal airflow, sound frequency, and sound intensity showed a positive direct relationship with the driving air pressure. While the natural calls of the studied species exhibit minimal frequency modulation, their larynges produced about an octave of frequency modulation in response to varying pulmonary pressure. Natural advertisement calls are produced near the higher extreme of frequency obtained in the laboratory and at a slightly higher intensity (6 dB). Natural calls also exhibit fewer harmonics than artificial ones, because the larynges were activated with the mouth of the animal open. The results revealed that treefrog larynges allow them to produce calls spanning a much greater range of frequencies than observed in nature; therefore, the simplicity of the calls is not due to a limited frequency range of laryngeal output. Low frequencies are produced at low intensities, however, and this could explain why treefrogs concentrate their calling at the high frequencies.

## 1. Introduction

Studies of calling in frogs have produced substantial insight to our understanding of sexual selection, speciation, and the evolution of communication systems [1–7]. The astonishing diversity of frog calls has inspired studies on the selective forces that drive their diversification and evolutionary history. Potential functional limitations have been examined in terms of energetic costs [8–10] and contractile performance of the muscles that push the air across the larynx [11–13], but additional insight might be gained from a better understanding of the vocal folds [14].

In relation to other vertebrates, most frogs produce fairly simple advertisement calls (with a few exceptions [15, 16]), but the extent to which such simplicity results from the calling apparatus or from the brain has not been determined yet. Frogs call by moving air from the lungs through the larynx into the mouth and vocal sac. A pair of vocal membranes passively vibrates producing sound as the air crosses the larynx [17–20]. These membranes are not muscular and they vibrate passively to produce sound [17, 21, 22]. Morphological studies have proposed that the posterior laryngeal constrictor muscle could be dedicated to controlling the tension of the vocal folds and the frequency of the calls [22, 23], but experimental tests have refuted the idea [19]. As a more general mechanism, however, pulmonary pressure (immediately upstream to the vocal folds) in excised larynges is directly correlated with the frequency of the acoustic output [17, 18, 24]. While a specific mechanism of frequency modulation has not yet been demonstrated in frogs, they should be able to vary the frequency of their calls by varying the pressure in their lungs.

In mammals, studies with excised larynges have found pulmonary pressure to correlate directly with both sound frequency and intensity [25]. This ties together the frequency and intensity of the acoustic output, but such a tie is almost completely overcome by control over muscular stretching and stiffening of the vocal folds, which allow for extensive decoupling between changes in call frequency and intensity [26]. If frogs lack the muscular control of vocal folds stretching and stiffening observed in mammals, frequency modulation in frog calls would be restricted to that produced by pressure changes. In addition, males would be locked into varying call intensity together with call frequency, such that low pulmonary pressures would result in soft, low-frequency calls and high pulmonary pressures would generate calls with high intensity and high frequency. This could generate a trade-off, because females of various species exhibit preferences for low frequency and high-intensity advertisement calls. Therefore, a male could call at low pulmonary pressures to produce sexy, low-frequency calls, but such calls would not be highly attractive for having low intensity. And if the male called at high pressures, then his calls would have high frequencies, which would hinder their attractiveness.

This study focused on three species of North American treefrogs that produce reduced frequency modulation in their advertisement calls relative to other frogs. I activated the larynges of euthanized specimens with airflow, analyzed their acoustic output, and compared them to field recordings to determine (1) if variation in pulmonary pressure could produce a wider range of frequencies than observed in natural advertisement calls, (2) if variation in the produced sound frequency is correlated with sound intensity, and (3) if natural calls match the maxima of frequency and intensity obtained in the lab.

## 2. Materials and Methods

### 2.1. Animals

I studied three species representing distinct clades within the treefrogs:* Hyla versicolor* (LeConte 1825),* Pseudacris streckeri* (Wright and Wright 1933), and* Acris crepitans* (Baird 1854). Their simple advertisement calls with little frequency modulation and their abundance in central Texas have directed the choice. This study was conducted under Texas collection permit SPR-0600-105 and Animal Utilization Protocol number 01021501 of the Animal Resource Center of the University of Texas.

**Figure 1:**
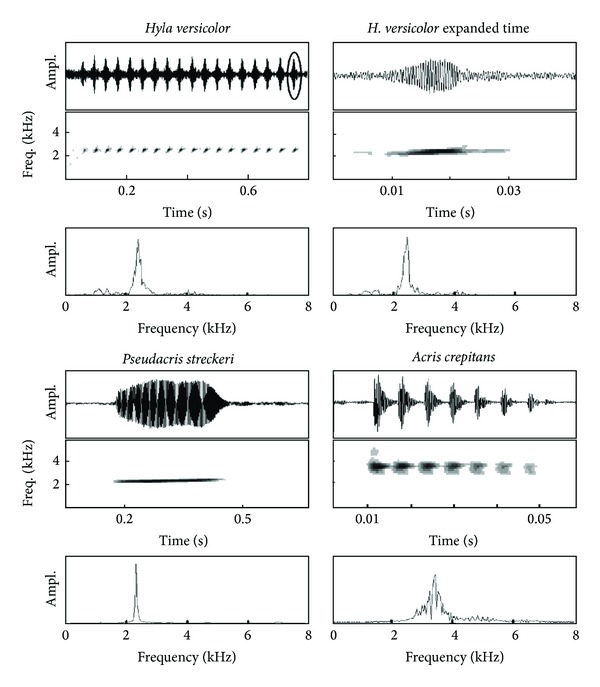
Advertisement calls of treefrogs from central Texas. Each note in the call of* H. versicolor* involves exhalation and inhalation (see Section 2.1). The pulses shown for* A. crepitans* are produced in a single exhalation.

The advertisement call of H. versicolor is a series of pulses [27] involving reinflation of the lungs before each pulse ([27]; Figure 1). The pulse was, therefore, the functional unit for comparison with the call of P. streckeri, which is composed of a single pulse. Acris crepitans produces complex advertisement signals composed of series of calls [28]. Each call can contain several pulses but these are not comparable to the pulses of H. versicolor because they are all produced along a single exhalation. The call, with its pulses, was the unit used for comparison with the other two species.

### 2.2. Field Recordings

Advertisement calls of ten calling males of* Hyla versicolor* were recorded at 20–22°C in May 2001 at the Stengl-Lost Pines Biological Station in Bastrop, TX. Calls of eight* Pseudacris streckeri* were recorded at 18–23°C in February 2001 and April 2002 at Gill Ranch in Austin, TX. And calls of eight* Acris crepitans* were recorded at 21–23°C in June 2001 and May 2002 at the Horse Thief Hollow Ranch in Austin, TX. Recordings were made with a shotgun microphone (Sennheiser ME-80) and a cassette recorder (Marantz PMD-420) at 1 m from the animal, in a position perpendicular to its longitudinal axis, at an angle of about 45° above the animal's coronal body plane. Recording tapes were calibrated for amplitude with tones recorded in the laboratory and in the field. The tones were produced with a custom-made device that was verified before each recording session with a precision sound pressure level meter (GenRad, model 1982). All recorded individuals were captured and brought to the laboratory for experimentation. Sounds were digitized at a 44100 Hz sampling rate with 16-bit resolution. Acoustic analyses were performed on ten calls per animal, using Sound Ruler 0.937b (http://soundruler.sourceforge.net). The recordings were high-pass filtered at 80 Hz to remove background noise and frequency measurements were obtained after fast Fourier transformation of the audio data using a Hanning window of 1024 samples. The sample sizes for individuals and calls were determined with base on the variability encountered in preliminary recordings used to test the method of calibration for sound pressure level.

### 2.3. Laryngeal Activation

**Figure 2:**
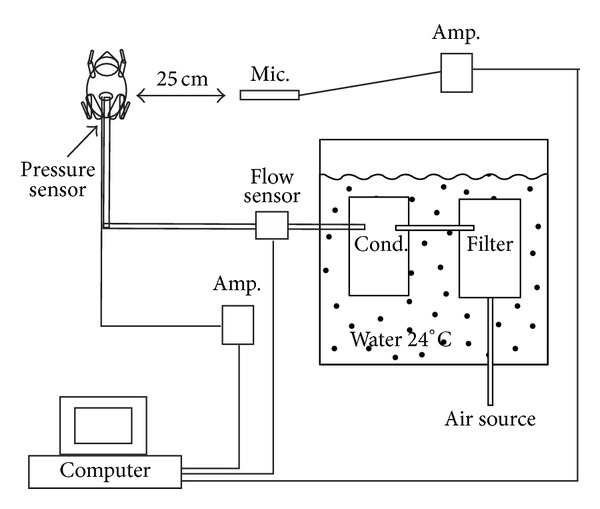
Experimental setup for laryngeal activation of frogs (mic. = microphone, amp. = amplifier, and cond. = condenser). Moist air is passed through the lungs of a euthanized frog and activates the larynx to produce sound. Pulmonary air pressure, airflow, and sound are recorded.

The animals were euthanized with benzocaine gel at 5% and immediately prepared for experimentation. The posterior tip of one lung was cannulated through an abdominal incision and the connection between lung and cannula was sealed with cyanoacrylate adhesive (Figure 2). The abdominal muscles and skin at the incision were also glued to the cannula sealing the body cavity. No difference was observed in relation to previous experiments in which both lungs were cannulated or in which the lungs, bronchi, heart, and liver were removed and the tip of the cannula was free in the body cavity. The cannula had an internal diameter of 2.4 mm. Endoscopic examination by attachment of a camera to the cannula revealed that the bronchial passages were wider than the cannula itself and no structure upstream from the vocal folds was likely to produce significant resistance to airflow, even in the smallest species.

A pressure sensor (Millar SPR-524, Houston, TX, USA) was inserted in the cannula with the tip protruding into the lung of the frog and the sensitive plate parallel to the airflow. The activation was performed with the mouth of the frog open, so that the pressure downstream from the vocal folds was equal to the ambient pressure. A thermistor-based airflow sensor (Cole-Parmer EW-32707-16, Vernon Hills, IL, USA; range 0–10 ± 1.5% l/min) was placed in the tubing 20 cm upstream from the cannula.

The air fed into the larynx was saturated with humidity at 24°C to prevent desiccation of the vocal folds. The air was blown by the experimenter and traveled 30 cm in tygon tubing to reach a filter and a condenser with a combined internal volume of 49.4 mL that were placed underwater at 24°C. The temperature drop produced condensation of the excess humidity and the air reached the tip of the cannula at 24 ± 0.2°C within the experimental airflow range. Preliminary trials with a pump blowing air at 64% humidity rendered the larynx dehydrated and aphonic in 40 and 80 s. A programmable steady-flow pump with wide pressure and airflow ranges, injecting air saturated with humidity at constant temperature, should improve the repeatability of the trials in this type of experiment, but it was not available for this study. Before each experiment, the seal of the tubing and its resistance to airflow were verified.

The calibration (error = 0.02 kPa at 3.2 kPa) and linearity (regression *r* ^2^ = 0.999, *n* = 10) of the pressure sensor were verified by immersion of its tip in a graduated water column at ten known depths for which theoretical values of pressure could be calculated. The airflow sensor was factory-calibrated and was verified (regression *r* ^2^ = 0.994, *n* = 5) by comparison with a fan-based airflow sensor in constant flow.

The entire preparation was installed in a custom made anechoic container to attenuate any echo or external noise. The container measured 0.76 × 0.76 × 1.1 m, with its exterior composed of plywood with a thickness of 12 mm, followed by 54 mm of high-density foam, another layer of 8 mm of plywood, and two more layers of 54 mm high-density foam. Pure tones played and recorded within the container resulted in sinusoidal waveforms with smooth amplitude envelope and less than 1.5% variation in amplitude among cycles at frequencies within 250 Hz to 20 kHz. An electret microphone (RadioShack 33–3003, USA) was placed at 25 cm from the preparation, perpendicular to the longitudinal axis of the animal. The microphone-recorder setup was previously calibrated for amplitude with a custom-built tone generator and a precision sound pressure level meter (GenRad, model 1982).

Following each trial of laryngeal activation by airflow, the preparation was inspected for air leaks and anatomical damage. The tubing system was tested for delays due to the distance between sensor placement and point of interest. Pressure and airflow were acquired at 11025 samples per second whereas sound was acquired at 44100 samples per second. Data were analyzed at intervals of 5 ms, averaging 1 ms of data for the pressure and airflow channels and taking the root of the mean squared (RMS) value for a period of 5 ms for the sound amplitude channel. In addition, a channel containing the frequency of the second harmonic was derived from the sound channel, through a series of Fourier transformations (FFT) of 1024 samples and 950 samples of overlap between successive FFTs. The second harmonic was used because it is the dominant frequency in the natural advertisement calls of all three species.

The speed at which pressure is varied in these experiments should be slower than the response of the slowest sensor, which was the airflow sensor. Preliminary experimentation revealed some inertia in the laryngeal response to changes in pressure. Data collection was, therefore, performed along gradual ascending and descending pressure ramps of dozens of seconds in duration. This condition is distinct from natural calls, in which the duration of each exhalation is less than 0.5 s in all the species studied. The experiments were concluded 40 minutes after euthanasia independent of the number of trials performed.

### 2.4. Data Analysis

Data from field recordings were examined with analyses of variance (ANOVA) and Bonferroni *t*-tests for post hoc pairwise comparisons. The results of artificial activation were analyzed separately for each species and dependent variable. Each analysis consisted of a nested analysis of covariance (ANCOVA), in which each trial was nested within individuals and pressure was the covariate.

While pressure could not be precisely controlled during the experiment, it was precisely monitored and it was varied over the entire range in which the larynx produced tonal sound. The manual manipulation should not, therefore, compromise the analysis of the relationships between pulmonary air pressure and acoustic output.

The residuals of such analyses were examined for autocorrelation. Any inertia in the response of the larynx to variations in lung pressure would compromise the independence of the residuals for subsequent data points. Nonindependence of the residuals does not compromise the fit of the regression, but it biases the calculations of significance. To remove the autocorrelation from each analysis, autocorrelation plots were produced and the minimum lag that rendered null autocorrelation was determined. The dataset was then reduced, and points were preserved at an interval equal to the null autocorrelation lag. The nested ANCOVA was then recalculated. All analyses were computed in SPSS (SPSS Inc., version 10).

## 3. Results

**Figure 3:**
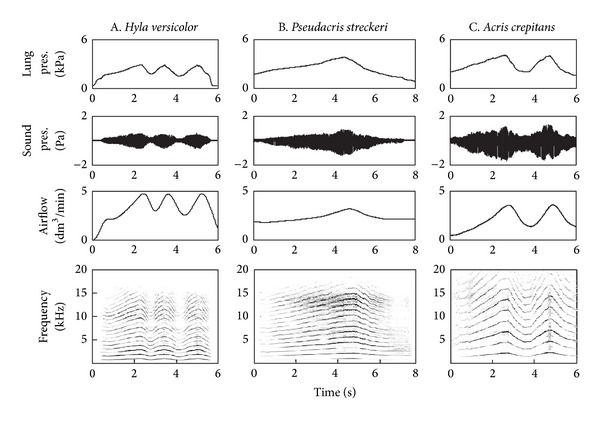
Laryngeal activation in three species of treefrogs with the mouth open. Pulmonary air pressure (lung pres.), airflow, sound pressure (sound pres.), are positively correlated with each other.

In all three species, variation in pulmonary pressure produced sound in a much wider range of sound frequencies than that encountered in the natural calls (Figure 3). Sound frequency, sound intensity, and airflow varied in direct correlation with pulmonary pressure. Natural calls are produced near the maximal sound frequencies obtained in the lab, at intensities about 6 dB higher than in the lab.

At very low pulmonary pressures, no sound was produced. At intermediate pressures, extensive frequency modulation was produced in proportion to pressure. At high pressures, the sound became noisy, losing its frequency structure but remaining intense, or the vocal folds retracted from the airflow and the sound output abruptly turned into soft hissing.

### 3.1. Physical Relationships

**Figure 4:**
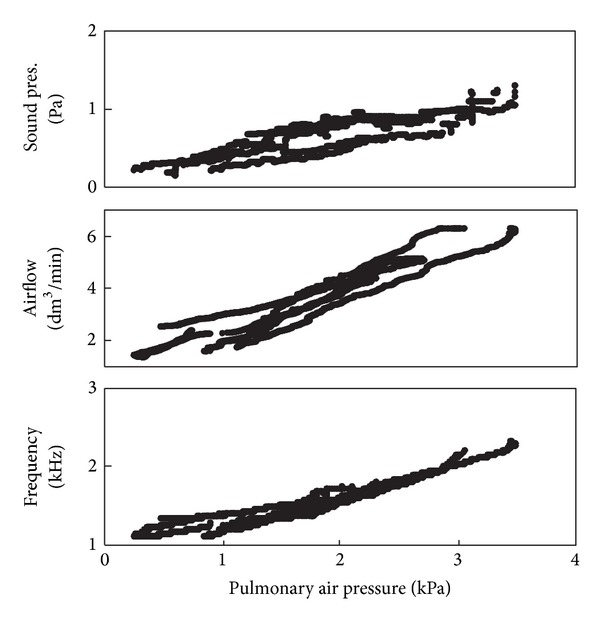
Relationship between pulmonary pressure and laryngeal airflow (*r* ^2^ = 0.88), sound pressure (*r* ^2^ = 0.72), or sound frequency (*r* ^2^ = 0.89) in one individual of* H. versicolor*. Sounds were produced artificially through laryngeal activation with the mouth open. Frequency refers to the second harmonic (natural dominant frequency).

**Figure 5:**
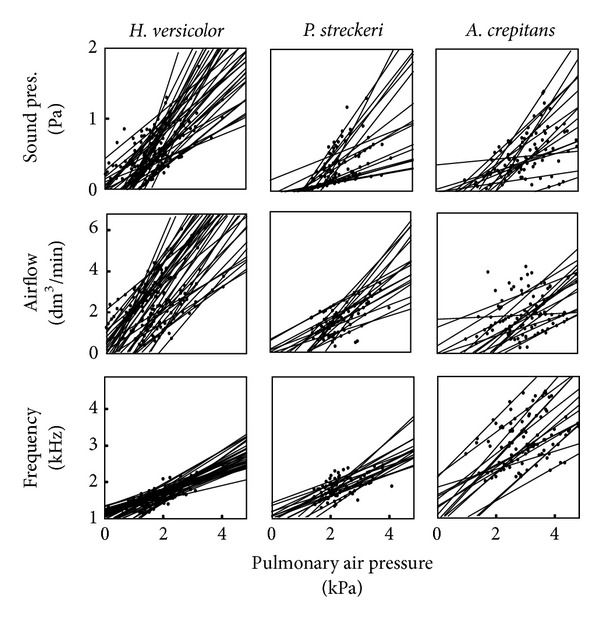
Laryngeal activation of the vocal folds of treefrogs. Regression lines are shown for each trial in eight individuals of* A. crepitans,* eight* P. streckeri*, and ten* H. versicolor.*

All measured variables presented positive direct relationships with each other within the ranges in which sound was produced (Figure 4). While the true relationships might not be exactly straight lines, such approximation was assumed in the subsequent analysis for simplicity [29]. The positive relationships between pulmonary pressure and sound amplitude, airflow, and frequency were verified for all three species (Figure 5). Nested analyses of covariance were computed to partition the variance observed in acoustic output among individuals and trials within individual, with pressure as the covariate.

The residuals of the analyses of amplitude, frequency, and airflow initially exhibited autocorrelation within lags of 0.83 s, 1.38 s, and 0.94 s for* H. versicolor*, 1.05 s, 0.72 s, and 0.72 s for* P. streckeri*, and 0.55 s, 0.50 s, and 0.55 s for* A. crepitans*, respectively. This can be interpreted as inertia of the laryngeal response to changes in pressure. Larger species showed longer autocorrelation lags, indicating greater inertia in larger larynges. The autocorrelation was removed from each analysis by reducing the data sampling rate (see Section 2.4) and the analyses were repeated.

### 3.2. Variation

In all three species, pressure had the most significant effect on the dependent variable, followed by individual and trial (Table 1). More than 92% of the variance in the data was explained by the nested ANCOVA for frequency and airflow in all three species. The analysis explained the greatest variance for* H. versicolor*, followed by* A. crepitans* and* P. Streckeri*.

To verify if the effect of individual in the analyses above could be explained by body size, the mean predicted value of each dependent variable was calculated for each individual at the pulmonary air pressure of 3 kPa. This pressure was chosen since it fell inside the range of pressures over which the vocal folds produced sound in all three species, and it resulted in fundamental frequencies close to natural ones. The variances of the values adjusted for 3 kPa of pulmonary pressure were examined, having body size as the covariate. The relationship was not significant for amplitude (ANCOVA *n* = 26, *F* = 0.12, and *P* = 0.73), airflow (*F* = 0.10; *P* = 0.76), or frequency (*F* = 0.74; *P* = 0.40). The effect of individuals is more likely due to variation in the elastic properties of the vocal system or maybe to mucus or condensation on the vocal membranes during activation. To verify if time since euthanasia could explain any variation among trials, the mean predicted values for each trial were also calculated. The relationship was not significant for amplitude (ANCOVA *n* = 107, *F* = 2.8, and *P* = 0.10), airflow (*F* = 2.9; *P* = 0.09), or frequency (*F* = 2.8; *P* = 0.10) indicating that experimentation did not produce relevant irreversible distension of tissues or other damages to the laryngeal structure.

### 3.3. Spectral Structure

**Figure 6:**
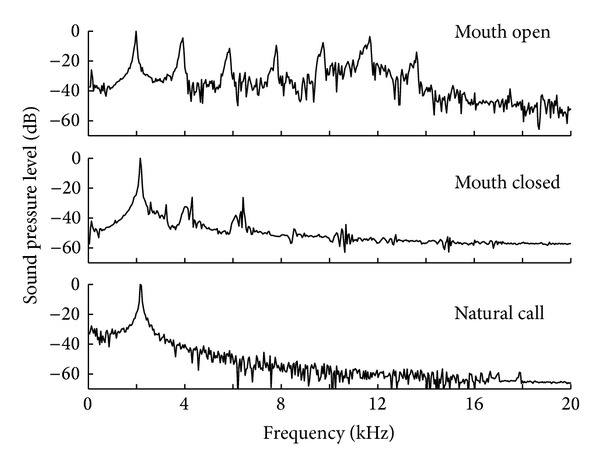
Spectral structure of natural and artificial calls in* Pseudacris streckeri*. Artificial calls were obtained with forced airflow through the passive larynx of a euthanized male frog, and the mouth was open or closed, and, in the latter case, the vocal sac would inflate. Notice the same fundamental frequencies but different distribution of energy in harmonics.

The sounds produced with laryngeal activation exhibited a pronounced harmonic structure (Figure 3). These extra harmonics were eliminated or greatly attenuated if the larynx was activated with the mouth closed and the vocal sac was allowed to inflate (Figure 6). Laryngeal activation with the mouth closed produced a spectrum that closely resembled that of the natural call.

**Figure 7:**
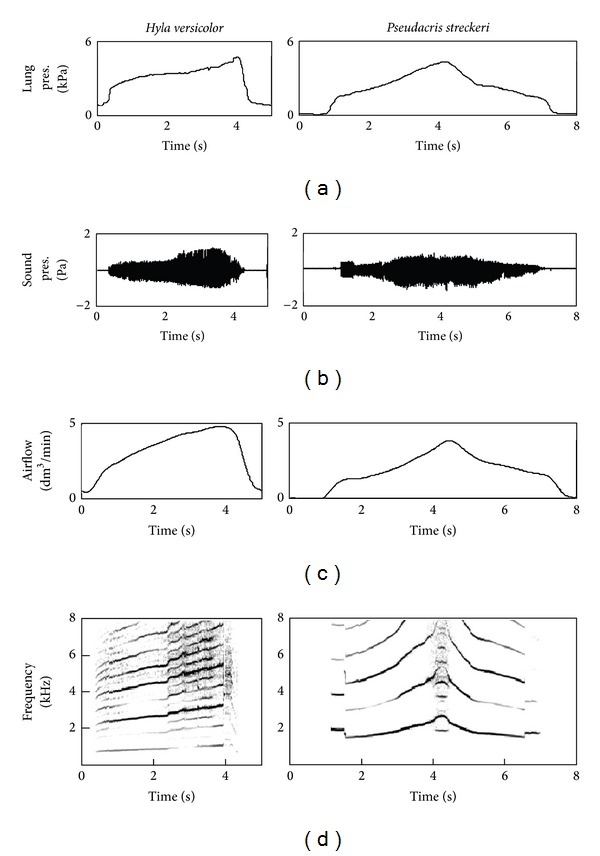
Occasional nonlinearities ((a), (b), (c), and (d)) in the acoustic response of the vocal folds of treefrogs to laryngeal activation.

At pressures close to the high and low limits of phonation of the larynx, nonlinearities were observed in the acoustic output of the larynx with the mouth of the frog open. The nonlinearities included sudden changes in fundamental frequency, sharp transitions between periodic and noisy sound, and doubling of the fundamental frequency through elimination of the odd harmonics (Figure 7). These observations were infrequent within most of the pressure range in which sound was produced.

### 3.4. Passive Acoustic Range

Laryngeal activation provided parameter estimates for the effect of lung pressure and laryngeal airflow on sound frequency, amplitude, and airflow and described the acoustic range of the passive larynx (Table 2). The observed acoustic ranges exhibit interesting relationships with size.* Hyla versicolor* is the largest in snout-to-vent length (mean ± SD) (41.3 ± 1.9 mm, *N* = 9) and vocal fold length (7.0 ± 0.7 mm, *N* = 5), measured as the distance between the articulation points of the arytenoid cartilages.* Pseudacris streckeri* is intermediate in body (34.2 ± 2.2 mm, *N* = 8) and vocal fold length (3.9 ± 0.4 mm, *N* = 5), and* A. crepitans* is the smallest (body 23.8 ± 0.9 mm, *N* = 7), but with a relatively large larynx (vocal fold length 3.3 ± 0.4 mm, *N* = 5). The larynges of the three species produced sound within approximately the same range of lung pressures. The larynx of* H. versicolor* reached higher airflows and sound pressures producing lower frequencies than the other two species. The larynx of* A. crepitans* reached higher intensities and much higher frequencies than* P. streckeri* at the same airflow.

### 3.5. Comparison with Live Advertisement Calls

**Figure 8:**
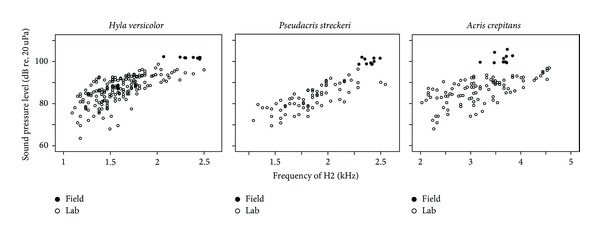
Comparison between natural advertisement calls (filled circles) and the ranges of acoustic output obtained through laryngeal activation (empty squares). Natural calls are placed near the high extreme of frequency and amplitude that the larynx can produce passively.

In* P. streckeri* and* H. versicolor*, natural advertisement calls are produced near the high extreme of frequency and amplitude of sound obtained in the laboratory through laryngeal activation (Figure 8). In* A. crepitans*, the natural calls are higher in frequency than the average sound obtained artificially; however, they are not at the high extreme as in the other two species.

The relationship between frequency and amplitude obtained during the forced-air experiments consistently underestimated the amplitude of natural calls for a particular frequency. This indicates that a relevant component of natural calling is lacking in artificial activation. This most likely involves the closed mouth with the inflation of the vocal sac. Previous studies have shown that the vocal sac of anurans radiates a large proportion of the acoustic output and that when the mouth is open, the output becomes detuned and weakened [30, 31]. These changes in output are possibly due to resonance of the vocal sac tissues or coupling between the vocal cords and the vocal sac in the closed system.

## 4. Discussion

### 4.1. Physical Relationships

The passive larynx of the hylids in this study exhibited a similar response to pulmonary pressure as previously observed in [18] for European* Rana* and [12, 17] for* Bufo*. The range of pressures over which the larynx produced sound was about the same obtained by Paulsen [18] (0.98 to 5.9 kPa) but much narrower than that employed by Martin [17] (up to 24.0 kPa), in experiments that did not necessarily reach the maximum functional limit of the larynx. The toads studied by Martin [17] range from 50 mm to 180 mm in snout-to-vent length, whereas the ranids studied by Paulsen [18] range from 70 mm to 110 mm. The extended functional range of the larynx of* Bufo* is therefore unlikely to be due to body size. It could derive, however, from the particular structural organization of the larynx of* Bufo*, having L-shaped vocal folds that lack a cranial ligament and having an extra pair of folds, posterior to the vocal folds [32].

Size accounted for some but not all of the functional variation in the species of the present study. The large species produced lower frequencies at a given pulmonary pressure than the small species. This was also the case in a broader analysis within the genus* Bufo* in which vocal fold mass was directly correlated with both sound frequency and body size [17]. The larynx of* A. crepitans* produced sound, however, over the same range of pressures as the other species and produced sound at similar intensities (Figure 8). The experimental data match the observation that natural advertisement calls of* Acris crepitans* are not less intense than those of the other two species [33] (Figure 8).

This study showed that sound amplitude and airflow exhibit a positive relationship with lung pressure in the larynx of hylids. Such relationship is also found in mammals but it can be masked by active control of vocal fold tension and length [29, 34, 35]. In songbirds, fundamental frequency is controlled and modified over an extensive range by the complex syringeal musculature whereas in nonsongbirds fundamental frequency is more strongly correlated with pressure in the interclavicular air sac then in the cranial thoraxic air sac or in the trachea [36, 37]. The functional description of the passive larynx in frogs provides a foundation for examination of potential mechanisms of active control, which should differ from those of other classes of vertebrates, given the extensive differences of laryngeal morphology observed among these groups [22, 38].

### 4.2. Passive versus Live Acoustic Range

Two major differences were observed between artificial and natural calls. First, the energy in artificial calls was distributed over a wide range of harmonics while natural calls emphasize the second harmonic. This mostly derives from the fact that the mouth of the animal was kept open during experimentation [30]. Trials with the mouth of the frog closed produced a pronounced decrease in energy in the higher harmonics and an increase in energy in the dominant frequency. This could be the result of acoustic coupling between the vocal folds and the vocal sac or filtering by the sac and amplification through tissue resonance (not cavity resonance) of the vocal sac as shown by Purgue [31]. Martin [12] and Capranica and Moffat [39] punctured the vocal sac of living* Bufo* and* Hyla versicolor*, respectively, and observed the same effect: calls had energy distributed over a wider range of harmonics than with the vocal sac intact. In addition, the experimental animals in the present study had the musculature relaxed, possibly altering the sound radiating properties of their body.

The second difference was that natural calls were slightly more intense than artificial ones produced at the same frequency. This could have occurred because, with the mouth open, tissue resonance in the vocal sac would be reduced, as the vocal sac would not inflate. Purgue [31] attached the carcasses of euthanized frogs to an artificial sound source and found that the tissues of* Hyla gratiosa* and* Pseudacris regilla* can resonate, producing an amplification of 15 dB in comparison to the sound recorded without the frog. Alternatively, the passive state of the vocal system could have diminished its output. Sound production by vocal folds is sensitive to precise alignment of their medial edges [40], to the lubrication of their surface [41], and to the positioning of the larynx. Live frogs might have feedback and control over all of these factors, allowing them to maximize the acoustic output of their vocal folds.

Laryngeal activation indicates that hylids could produce frequency modulation by varying the contraction strength of the trunk muscles that raise pulmonary pressure. Field provides evidence that such compromise is also relevant to life frogs. Males of* Acris crepitans* have been shown to lower the frequency of their calls during male-male interactions, producing calls with reduced amplitude [42]. In addition, when males of* Eleutherodactylus coqui* are making their very first calls of the night, there is often an increase in the frequency and amplitude of the call [43]. Martin [17] pointed out that the same relationship can be observed in the very first notes of some calls in* Bufo*. Dudley and Rand [44] estimated vocal sac volume and laryngeal airflow in* Engystomops pustulosus* through video analysis and found a correlation between airflow and frequency. They suggested that frequency modulation in* E. pustulosus* could be explained by variation in airflow due to contraction strength of the body wall.

The vocal folds of frogs lack embedded muscles that would allow for the extensive control of frequency found in most mammals [22, 32]. Mechanisms for stretching of the insertion points of the vocal folds could allow for a less direct control of sound frequency, as observed in vespertilionid bats [45–47].

In response to acoustic stimulation, males of* Leptodactylus albilabris* are able to change the dominant frequency of their calls by as much as 400 Hz and the intensity of their calls by up to 16 dB [48]. Changes in call frequency in this species are not directly correlated to changes in call intensity. Anatomical evidence suggests that anurans could control frequency modulation through contraction of the posterior laryngeal constrictor muscles that are external to the larynx [22], but this hypothesis has not been confirmed experimentally [19].

### 4.3. Nonlinearities at the Vocal Folds

Fee and colleagues [14] studied the vibration of the vocal membranes of zebra finches (*Taeniopygia guttata*) and found nonlinear behaviors in response to gradual changes in bronchial pressure. These sharp transitions were present in the birds' songs, indicating that some of the vocal complexities derive from passive changes in the vibration mode of the vocal membranes. Similar nonlinearities have also been described in other vertebrates including humans [49] and the frog* Amolops tormotus* (Ranidae) that produces highly variable and complex calls which include ultrasound [50]. In the present study, nonlinear behavior of the vocal folds was observed mostly at extremely low or high pressures and they seem not to be present in the advertisement calls of the species examined. In a few cases, however, nonlinearities were observed at intermediate pressures. Those might result from individual variation in membrane stability or they might have been caused by displacement of mucus or condensation on the vocal membranes during experimentation.

## 5. Conclusions

The vocal apparatus of treefrogs is capable of producing sound at a much broader range of frequencies than that found in their natural advertisement calls. A direct relationship between pulmonary pressure, airflow, sound amplitude, and frequency determines the output of the passive larynx. In case treefrogs lack other mechanisms of frequency modulation, the production of low frequencies would require reducing the intensity of the calls. Future work should screen for mechanisms of frequency modulation in frog species that produce extensive frequency modulation and verify the effectiveness of any such mechanisms in treefrogs.

## Figures and Tables

**Table 1:** Variance in laryngeal output partitioned among pressure (covariate), individual, and trial within individual, using analyses of covariance. Nine separate analyses address three species and three dependent variables: amplitude, airflow, and frequency.

Variables		Species					
Dependent	Independent	*H. versicolor *		*P. streckeri *		*A. crepitans *	
		*F*	% Var.^a^	*F*	% Var.^a^	*F*	% Var.^a^
Amplitude	Pressure	513∗∗∗	43	55∗∗∗	22	131∗∗∗	26
	Individual	13∗∗∗	32	17∗∗∗	53	12∗∗∗	48
	Trial (indiv.)	4∗∗∗	11	1 n.s.	0	3∗∗∗	11
	Error		14		25		15

Airflow	Pressure	1037∗∗∗	36	515∗∗∗	40	214∗∗∗	20
	Individual	16∗∗∗	49	3∗	30	4∗∗	37
	Trial (indiv.)	11∗∗∗	11	19∗∗∗	25	17∗∗∗	36
	Error		4		5		7

Frequency	Pressure	851∗∗∗	58	847∗∗∗	36	420∗∗∗	23
	Individual	6∗∗∗	19	7∗∗∗	46	4∗∗	26
	Trial (indiv.)	6∗∗∗	16	24∗∗∗	16	31∗∗∗	45
	Error		7		2		6

^a^% var. = percent of the variance explained by the independent variable. Significance (not corrected): ∗∗∗*P* < 0.001, ∗∗*P* < 0.01, and ∗*P* < 0.05; n.s. = not significant.

**Table 2:** Relationship between pressure differential (PD) across the larynx and sound frequency (FR), laryngeal airflow (AF), or sound pressure (SP) for three species of treefrogs as fit with straight lines.

Species	Frequency (Hz)	Airflow (dm^3^/min)	Sound pressure (Pa)
*H. versicolor *	FR = 337PD − 33153	AF = 1.573PD − 159.467	SP = 0.415PD − 42.202
*P. streckeri *	FR = 345PD − 33924	AF = 0.889PD − 90.689	SP = 0.226PD − 23.087
*A. crepitans *	FR = 600PD − 59450	AF = 0.799PD − 81.504	SP = 0.254PD − 26.040
